# Surviving on low-energy light comes at a price

**DOI:** 10.7554/eLife.82221

**Published:** 2022-09-02

**Authors:** Elisabet Romero

**Affiliations:** 1 Institute of Chemical Research of Catalonia, The Barcelona Institute of Science and Technology Tarragona Spain

**Keywords:** photosynthesis, photochemistry, cyanobacteria, photosystem II, far-red light, low energy light, Other

## Abstract

Two species of photosynthetic cyanobacteria can thrive in far-red light but they either become less resilient to photodamage or less energy efficient.

**Related research article** Viola S, Roseby W, Santabarbara S, Nürnberg D, Assunção R, Dau H, Sellés J, Boussac A, Fantuzzi A, Rutherford AW. 2022. Impact of energy limitations on function and resilience in long-wavelength photosystem II. *eLife* **11**:e79890. doi: 10.7554/eLife.79890.

Life on Earth as we know it depends on photosynthesis. In this process, plants, algae and cyanobacteria use solar energy to convert water and carbon dioxide into the oxygen we breathe and the glucose that fuels many biological processes (Blankenship, 2002).

The photosynthetic machinery is made from an intricate and complicated collection of protein complexes containing mainly chlorophyll *a* molecules and carotenoid molecules. Several of these complexes work together in a synchronised fashion to accomplish an amazing feat: to split water molecules (one of the most stable molecules on earth) to extract electrons and produce oxygen, and to transfer electrons to quinone molecules so that photosynthesis can continue. The efficiency of this initial energy-conversion step determines the outcome of the whole process.

For many years it was thought that the red light absorbed by chlorophyll *a* (which has a wavelength of 680 nanometres) was the lowest-energy light that could be used to drive oxygenic photosynthesis while minimising the damage caused by high levels of light (Rutherford et al., 2012). However, previous research has shown that some cyanobacteria living in shaded environments can thrive in far-red light, which is close to the limit of what we can see: this light is lower in energy than red light because its wavelength is longer (720 nanometres) (Figure 1). These cyanobacteria contain chlorophyll *d* and chlorophyll *f* in addition to chlorophyll *a*, but they can still perform the same reactions as organisms that contain only chlorophyll *a* (Miyashita et al., 1996; Renger and Schlodder, 2008; Gan et al., 2014; Nürnberg et al., 2018; Davis et al., 2016). So far, however, it has not been clear if these species pay a price in terms of resilience to photodamage or energy conversion efficiency.

**Figure 1.**
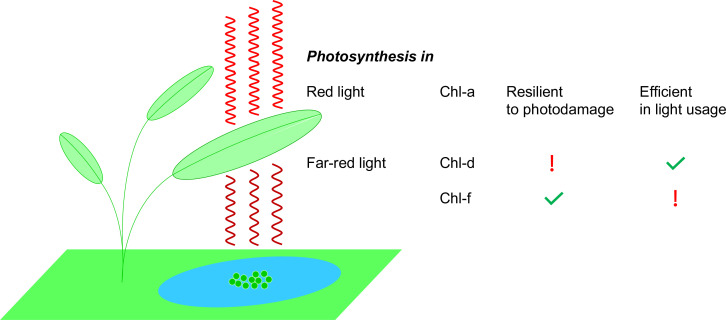
Photosynthesis in red light and far-red light. Plants, algae and cyanobacteria use a molecule called chlorophyll *a* (Chl-a) to absorb red light to power the process of photosynthesis. Studies have shown that Chl-a is resilient to photodamage and is efficient in using light energy. Some cyanobacteria (green circles in the blue pond; not to scale) have adapted to their darker environments by using different chlorophyll molecules – chlorophyll *d* (Chl-d) and chlorophyll *f* (Chl-f) – to absorb far-red light (which is less energetic than red light). However, the use of these molecules comes at a price: Chl-d organisms are energy efficient but they are not resilient to photodamage; Chl-f organisms, on the other hand, are not energy efficient but they are resilient to photodamage.

Now, in eLife, Stefania Viola and A William Rutherford of Imperial College London and colleagues – who are based at Imperial, the CNR in Milan, the Free University of Berlin, Sorbonne University and CEA-Saclay – report new insights into far-red photosynthesis (Viola et al., 2022). The team studied two species of cyanobacteria that use far-red photosynthesis: *Acaryochloris marina*, which lives in shaded environments, and *Chroococcidiopsis thermalis*, which lives in variable light conditions and can switch between standard photosynthesis and far-red photosynthesis depending on the light energy. To assess whether these organisms have the same resilience to photodamage and energy conversion efficiency as organisms that contain only chlorophyll *a*, Viola et al. investigated many aspects of the first initial energy-conversion step in photosynthesis (as described above). This included measuring the amount of oxygen generated, as well as the quantity of reactive oxygen species produced, which can lead to photodamage.

This revealed that both species of cyanobacteria produced comparable amounts of oxygen to chlorophyll *a*-only cyanobacteria. The photosystem II of *A. marina*, which contains 34 chlorophyll *d* molecules and just one chlorophyll *a* molecule, was highly efficient, but also produced high levels of reactive oxygen species when exposed to high-light levels, making it less resilient to photodamage. In contrast, when *C. thermalis* – which contains four chlorophyll *f*, one chlorophyll *d* and 30 chlorophyll *a* molecules – was grown in far-red light it produced fewer reactive oxygen species, but was also less energy efficient.

Viola et al. present a detailed picture of how these organisms have adapted to the low-light conditions specific to their environments and the associated costs of their traits. A better understanding of how photosynthetic organisms make use of low-energy light could help scientists to engineer far-red photosystems into algae or plants containing only chlorophyll *a*, thereby enhancing their use of sunlight and improving crop yields (Chen and Blankenship, 2011). Moreover, in the future, artificial systems could be designed to use low-energy light to generate solar fuels.
